# Protecting health care workers in the front line: Innovation in COVID-19 pandemic

**DOI:** 10.7189/jogh.10.010357

**Published:** 2020-06

**Authors:** Zihui Tan, Deborah Wen Shi Khoo, Ling Antonia Zeng, Jong-Chie Claudia Tien, Aaron Kwang Yang Lee, Yee Yian Ong, Miqi Mavis Teo, Hairil Rizal Abdullah

**Affiliations:** Department of Anesthesiology, Division of Anesthesiology and Perioperative Medicine, Singapore General Hospital, Singapore

The COVID-19 pandemic has now infected almost 700 000 people, killing more than 30 000 people. Although Singapore was previously able to control the rapid rise in daily cases through tight quarantine, rapid contact tracing and strict social distancing measures, our health care institutions are now facing a second surge from imported cases. The protection of health care workers (HCWs) is vital in continuing patient care in health care systems that are currently challenged by the pandemic, but also important in ensuring they do not spread the virus. In our country, there are no guidelines or unified practices as to the degree of HCW protection required for performing routine throat swabs. A unique feature of many testing venues in Singapore is that they are outdoors with the average of 30°C tropical weather, rendering the prolonged use of conventional personal protective equipment (PPE) or full-body protection uncomfortable. The widespread incidence and expected protracted duration of the COVID-19 pandemic has also prompted concerns for minimising the use of PPE especially for high-volume or brief procedures with a short duration of high-risk patient contact, such as throat swabbing. We offer this invention as a versatile component of a modular system that can be adapted to several situations and clinic setups. HCWs no longer have to change their disposable face shield, cap and gown between patients. This has allowed us to conserve our current PPE supply. In time when testing may be carried out more extensively in community settings, we hope that this would ease logistic difficulties in streamlining the need to test a heavy caseload.

It has been close to three months since the first COVID-19 case was diagnosed in Singapore [1]. The COVID-19 pandemic has now infected almost 700 000 people, killing more than 30 000 people [2]. Although Singapore was previously able to control the rapid rise in daily cases through tight quarantine, rapid contact tracing and strict social distancing measures, our health care institutions are now facing a second surge from imported cases. Given our country’s unique geographical location, and inherent lack of natural resources and raw materials, we are ultimately dependent on open trade borders to maintain our supply chain. As more countries start to implement travel and border restrictions and in various countries; a total lockdown, this will compromise our ability to maintain a comfortable supply of personal protective equipment.

The protection of health care workers is vital in continuing patient care in health care systems that are currently challenged by the pandemic, but also important in ensuring they do not spread the virus. In Hubei, China more than 3000 health care workers have been infected and in Italy 20% of responding health care workers were infected [3,4]. Our Singapore public health institutions have had 8 cases of COVID-19 infections among staff [5]. Singapore also depends on an intensive testing programme with one of the highest rates of testing globally at 6800 tests per million people as of 25 March 2020 [6].

Since January, our emergency department colleagues have been at the frontline battling the surge in attendance due to the pandemic. Throat swabs of suspected patients from the community are taken in a designated fever area (**Figure 1**). Up to 70 patients are seen daily in this area and the number will only increase. Although full personal protective equipment (PPE) is provided, concerns regarding PPE wastage and the need for conservation have surfaced. This is due to the continuing rapid increase in the number of patients seen in the community.

**Figure 1:**
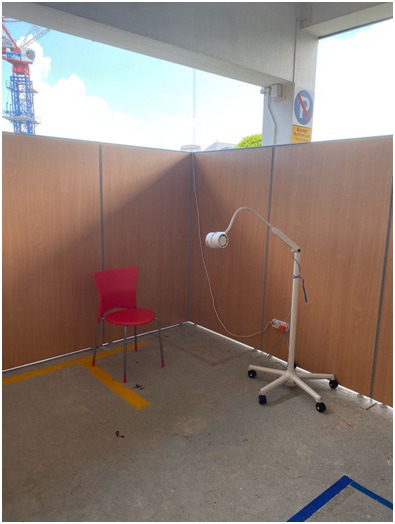
Designated fever area for throat swab.

Another important consideration is the proximity of the health care worker to the suspected patient especially when the patient sneezes, coughs or gags. Whilst nasal swabs were initially taken, there has been a shortage of these. Therefore, we have moved to throat swabs for testing. One study showed that throat swabs have a lower pick up rate as compared to nasal swabs [7], hence the importance of proper swabbing technique to accurately diagnose COVID-19. By providing better protection for the health care workers (HCW), we hope to reduce the incidence of false negatives and hence false assurance.

## SG-SAFE (SINGAPORE SWAB ASSURANCE FOR EVERYONE)

Together with a local bioengineering company, The Biofactory Pte Ltd, we proposed a screen between the patient and the HCW that fits the following criteria:

- Protect HCW from droplet ± aerosol contamination,
- Clear barrier for visualization,
- Light source to visualize oropharynx,
- Good dexterity, ie able to use both hands for tongue depressor and swab,
- Easy to clean, meets local infection control standards,
- Mobile,
- Easy for storage,
- Dual functionality, ie, it can be inverted to contain the patient as well.

**Figure 2:**
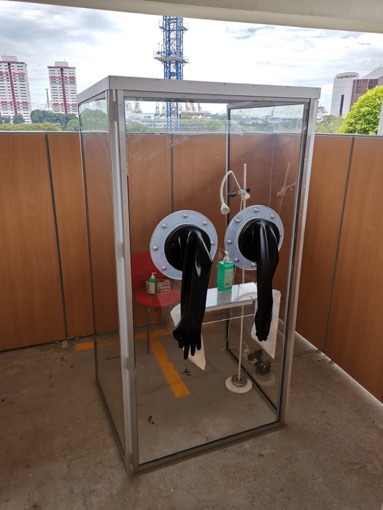
SG-SAFE – Singapore Swab Assurance for Everyone.

The first prototype was subsequently tested and used in the Emergency department (**Figure 2**). HCW expressed increased confidence for personal safety despite the high number of suspected patients seen daily.

More importantly, HCWs no longer have to change their disposable face shield, cap and gown between patients. This has allowed us to conserve our current PPE supply in view of the potential supply shortage if the pandemic continues for a prolonged period of time.

Innovation in health care is itself difficult, balancing the competing concerns for patient and operator safety, infection control, resource conservation and cost. The current pandemic has exacerbated these restrictions, but ironically made it all the more urgent that efficient and innovative solutions are sought out to address surging patient loads and high infectivity.

Reported examples of innovation in this pandemic range in scale from individuals repurposing scuba diving masks with 3D-printed “Charlotte valves” [8] to vacuum cleaner and automotive manufacturers producing ventilators [9,10].

Testing suspected patients is a cornerstone of epidemiologic control of this outbreak. Various devices have been described, from the South Korean “phone booth” [11] to simple plastic shields shown in media from the UK and Taiwan [12] The aim of these devices is both to contain infection as well as protect a HCW exposed to tens to hundreds of suspect cases. Some of the features of these existing devices are seen in **Table 1**.

**Table 1:** Advantages and disadvantages of current devices used during testing of COVID-19 patients

Feature	Advantage	Disadvantage
South Korean “phone booth”	Negative pressure environment contains aerosols and airborne particles generated by patient	Intensive sanitisation required between patients for reusable parts eg, semi-permanent gloves
	Virtually zero contact between operator and patient	Power source/generator required to maintain negative pressure environment; difficult to adapt for community/field use
Plastic shield with circular arm holes	Easily manufactured, reproducible, portable	No light source
		Plastic must be able to withstand high-level disinfection or be prone to degradation
		Limited protection for HCW whose limbs and hands must be enclosed in separate PPE
PPE alone / with added plastic face shield	Highly portable, versatile to any clinical situation	Resource-intensive if single-use per patient

HCW – health care worker, PPE – personal protective equipment

In our country, there are no guidelines or unified practices as to the degree of HCW protection required for routine testing. A unique feature of many testing venues in Singapore is that they are outdoors with the average of 30-degree-Celsius tropical weather [13], rendering the prolonged use of conventional PPE or full-body protection uncomfortable.

The widespread incidence and expected protracted duration of the COVID-19 pandemic has also prompted concerns for minimising the use of PPE especially for high-volume or brief procedures with a short duration of high-risk patient contact, such as throat swabbing. While single-use items have been the erstwhile gold standard of HCW protection and reduction of cross-contamination, we recognise that this is also reliant on supply chains and in many cases overseas manufacturer capacities (that are themselves subject to stresses of the pandemic in their own countries).

We offer this invention as a versatile component of a modular system that can be adapted to several situations and clinic setups. In time when testing may be carried out more extensively in community settings, we hope that this would ease logistic difficulties in streamlining the need to test a heavy caseload.

Our innovation allows for a reversal of the traditional model where an infectious patient is in a negative pressure room, as this requires significant time and labour to disinfect the room between patients. By allowing the health care worker to be protected inside and the patients to pass through outside in an outdoor setting, it will allow for much shorter times between patients and thus be able to rapidly collect swabs for large numbers of patients if the outbreak worsens.

Difficulties encountered in the production of this device were exacerbated by the rapid evolution of management strategies for the pandemic. As a relatively “unknown enemy”, the requirements of infection control policy and organisational directives were developing as practitioners on the ground sought to counter practical challenges such as the heat and fatigue from rapid and repeated donning and doffing of PPE.

Ready access to bioengineering expertise enabled the rapid production of a prototype. The use of technology to visualise and transmit ideas allowed for multiple practitioners to give their input remotely. Video-conferencing platforms allowed for immediate remote previewing of a physical prototype, while mobile messaging facilitated rapid transfer of images and feedback to and from multiple parties and stakeholders. More importantly, tele-communication also reduced the need for physical meetings and also prevented spread of infection by allowing for physical distancing without hampering or slowing the innovation process.

Emergency grants would accelerate device production in view of the ongoing pandemic, similar to the additional publication of COVID-19 related research in medical literature. An extraordinary time in human history calls for special measures to match the needs of a shifting and transforming battleground. As various industries turn their efforts to addressing the needs of health care, those on the ground should be equipped to contribute their first-hand expertise by all means possible.
